# Association of postmortem mitragynine positivity with fatal drug toxicity in polysubstance exposures—a restrospective analysis

**DOI:** 10.3389/fpubh.2026.1923369

**Published:** 2026-10-30

**Authors:** Armiel Suriaga, Ruth M. Tappen, Christopher R. McCurdy, David Newman, Maria C. Mejia, Lewis Nelson, Oliver Grundmann

**Affiliations:** 1 Christine E. Lynn College of Nursing, Florida Atlantic University, Boca Raton, FL, United States; 2 College of Pharmacy, Department of Medicinal Chemistry, University of Florida, Gainesville, FL, United States; 3 Charles E. Schmidt College of Medicine, Florida Atlantic University, Boca Raton, FL, United States

**Keywords:** drug intoxication, drug toxicity-related death, kratom, mitragynine, polydrug use

## Abstract

**Introduction:**

Kratom (*Mitragyna speciosa* Korth.) and kratom-related product use has increased in the United States, yet their contributions to drug-related death at the population level remain poorly defined. There is no established link between the amount of kratom ingested and the cause of toxic adverse effects, and evidence regarding their potential association with drug toxicity-related death is limited.

**Objective:**

To evaluate the association between kratom (mitragynine) exposure among decedents and assess its role in polysubstance-related mortality.

**Methods:**

A statewide retrospective cross-sectional analysis of 61,234 decedents with postmortem toxicology data. Kratom, as indicated by mitragynine measured in postmortem blood, was classified as present or absent. This is not a quantitative measure. The primary outcome was drug toxicity-related death (DTRD), determined by medical examiners. Multivariable logistic regression models estimated adjusted odds ratios (aOR), controlling for demographics, comorbidities, and co-exposure to other substances. Effect modification by sex and sensitivity analyses incorporating opioid exposure burden were performed.

**Results:**

Mitragynine was associated with increased odds of DTRD (aOR ~ 5–6). Among mitragynine-detected decedents, 85% of cases were classified as DTRD, and >90% had polysubstance exposure. Opioids showed the strongest association with DTRD, followed by cocaine and other stimulants, while benzodiazepines were modestly associated. Alcohol and cannabis were inversely associated with DTRD. Male sex and polysubstance exposure were associated with higher odds of DTRD. Older age and selected comorbidities (cardiac, respiratory, and renal disease) were associated with lower observed odds.

**Discussion/Conclusion:**

In this statewide cohort, postmortem mitragynine-positive cases were associated with increased odds of DTRD, almost entirely in the context of polysubstance use, while Mitragynine-positive DTRDs without other documented substances were uncommon. The association between postmortem mitragynine-positive cases and DTRD supports its inclusion in toxicological surveillance and risk assessment frameworks.

## Introduction

Kratom (*Mitragyna speciosa*), a tropical plant native to Southeast Asia, has gained increasing popularity in the United States and other Western countries over the past decade (41). An estimated 1.7 million kratom users have been reported in 2021 (46). However, this number is likely underestimated based on product availability and sales figures (the American Kratom Association estimated >15 million users in 2019). A kratom industry-aligned report estimated approximately 10 to 15 million kratom users based on use patterns and import volume (1). Kratom’s primary active alkaloid, mitragynine, produces dose-dependent stimulant and opioid-like effects. Kratom is commonly used for self-management of pain, mood disorders, and opioid withdrawal (2–5). The widespread availability and increasing diversity of kratom products, combined with perceptions of being a “natural” and therefore safer alternative to opioids, have contributed to its growing use across diverse populations (6–8). Furthermore, products marketed as kratom may actually contain the semi-synthetically derived 7-hydroxymitragynine, a metabolite of mitragynine.

Despite this expansion, the safety profile of kratom remains incompletely characterized. Preclinical and pharmacologic studies demonstrate that mitragynine, the most abundant alkaloid, acts as a partial-biased μ-opioid receptor agonist and interacts with adrenergic and serotonergic systems (9, 10, 43, 47, 48). These properties raise important concerns regarding its potential for toxicity, dependence, and clinically significant drug–drug interactions. Increasing reports of adverse outcomes, including respiratory depression, seizures, and fatal overdose, have been documented, often in the context of polysubstance exposure (11–13). The diversity of kratom formulations and chemical composition of products differs from processed leaf material to concentrated extracts and, increasingly, synthetic or semi-synthetic alkaloid formulations, especially 7-hydroxymitragynine products. With increased alkaloid concentration and variable chemical composition comes a potential risk for toxicity across product types (14, 15, 41). However, much of the existing evidence is derived from case reports, poison control center data, or small observational studies, limiting the ability to quantify population-level risk. Case reports are subject to selection and publication bias and often lack detailed information on dose, product formulation, and co-exposures; while poison control center data, such as those collected by the American Association of Poison Control Centers, rely on voluntary reporting and self-reported exposures, which may be incomplete or inaccurate (13, 14, 16). Both data sources are further limited by frequent poly-substance use and the absence of standardized toxicological confirmation, restricting causal inference and generalizability.

In 2025, a 1,200% increase in kratom exposures, with the important recognition of non-classification of product, and the introduction of synthetic alkaloids (i.e., 7-hydroxymitragynine, mitragynine pseudo indoxyl, MGM-15), has been reported to the U. S. Poison Center, from 258 in 2015 to 3,484 in 2025 (17–19, 43). In our previous study, we reported 484 cases of Drug-Intoxication Related Mortality (DIRM) from kratom exposures in Florida from 2020 to 2021 (20). A major gap in the literature is the association between kratom use and postmortem mitragynine-positive cases in drug toxicity-associated mortality risk. While postmortem mitragynine positivity can occur from the use of kratom leaf material, it can also be present with the consumption of 7-hydroxymitragynine and other products marketed as “kratom.” The literature suggests that postmortem mitragynine positivity is primarily associated with co-occurring high-risk substance use (12). This distinction is particularly important given the high prevalence of polysubstance use among individuals who consume kratom, including concurrent use of opioids, stimulants, and other psychoactive substances.

While associated drug toxicity is a multifactorial process, previous studies have attempted to explain the mechanism of Drug Toxicity-Related Death or DTRD involving kratom (21, 22). Kratom intoxication reflects a dose-dependent convergence of toxicokinetic and toxicodynamic processes driven primarily by its indole alkaloids, mitragynine and 7-hydroxymitragynine (23, 43). Following ingestion, mitragynine is absorbed and undergoes extensive metabolism, including hepatic biotransformation, although available evidence regarding its metabolic pathways and disposition remains limited (45). Hepatic function is an important determinant of drug clearance and systemic exposure, and hepatic dysfunction can alter drug pharmacokinetics, potentially modifying the toxicity risk of co-administered substances (44). Mitragynine causes drug interactions by time-dependent inhibition of CYP3A, primarily in the intestine, thereby reducing first-pass metabolism and increasing the bioavailability of co-administered substances (24, 25). CYP3A is a subfamily of enzymes within the cytochrome P450 system that are responsible for metabolizing many substances in the body; even more than half of all prescribed drugs, such as opioids (26). Co-exposure of kratom with other drugs heavily metabolized by CYP3A could cause clinically relevant and potentially serious drug interactions (25).

At the molecular level, kratom alkaloids act as partial agonists at μ-opioid receptors while also interacting with adrenergic and serotonergic systems, producing a biphasic profile in which lower doses yield stimulant-like effects and higher doses induce opioid-like central nervous system depression (7, 27). With escalating exposure, receptor-mediated signaling becomes dysregulated, leading to impaired neuronal excitability, respiratory depression, and altered autonomic tone. Concurrently, hepatic metabolic burden and oxidative stress responses may precipitate hepatocellular injury, while systemic effects, including acid–base disturbances and cardiovascular instability, emerge as compensatory mechanisms fail (13, 28, 29). However, to date, there is no established link between the amount of kratom ingested and the severity of toxic adverse effects, as the concentration of specific alkaloids can be highly variable among products. In recent years, products containing a predominance of single kratom alkaloids like 7-hydroxymitragynine or semi-synthetic derivatives have entered the market. They are often sold as “kratom” despite differing from the native leaf material in composition and potentially containing other substances or adulterants. The FDA in 2025 recommended that 7-hydroxymitragynine be placed in CSA Schedule 1, and several states have banned the sale of 7-hydroxymitragynine, while natural leaf kratom products remain available.

Epidemiologic and clinical data further demonstrate that these processes are amplified by interindividual variability and co-exposures, culminating in a spectrum of toxicity ranging from mild sedation to life-threatening multisystem dysfunction (30). Collectively, toxicity associated with high doses of kratom alkaloids may present in a nonlinear, systems-level failure in which pharmacologic receptor engagement, metabolic activation, and organ-specific vulnerability interact to determine clinical severity.

In this study, we evaluate kratom exposure and its association with DTRD in a large, population-based sample. We specifically examine whether this association persists after adjustment for demographic characteristics, comorbid conditions, and polysubstance use. We hypothesize that kratom exposure is associated with increased odds of DTRD in a polysubstance use setting. By clarifying this relationship, our findings aim to inform clinical risk assessment, improve screening practices, and guide public health responses to the evolving landscape of substance use.

## Methods

### Study design and setting

We conducted a retrospective cross-sectional study of deaths investigated within the Florida Medical Examiners Commission (MEC) Drugs Identified in Deceased Persons reporting system from 2020 through 2023. The study focused on the association between toxicologically documented mitragynine exposure, used as an analytical marker of kratom exposure, and drug toxicity–related death (DTRD) among decedents with at least one substance detected on the available toxicology record.

The analytic dataset comprised 61,234 decedents across all 67 Florida counties. These cases represent deaths for which relevant toxicology information was available and reported through the MEC system and should not be interpreted as a census of all deaths occurring among Florida residents.

The Florida Atlantic University Institutional Review Board determined that the study constituted nonhuman-subjects research.

### Data source and case selection

Data were obtained from the Florida Department of Law Enforcement (FDLE) Medical Examiners Commission (MEC) Drugs Identified in Deceased Persons reporting system. Under Florida Statute §406.11, Florida medical examiners investigate deaths occurring under specified circumstances, including suspected poisoning or toxic exposure, accidents, suicide, criminal violence, sudden or unexpected deaths, unattended deaths, deaths in custody, and other suspicious or unusual deaths. Medical examiners may obtain laboratory examinations considered necessary to determine the cause and manner of death.

Florida Medical Examiners Practice Guidelines recommend toxicology testing when the circumstances of death and available history indicate a reasonable probability of intoxication and appropriate specimens are available. Accordingly, inclusion in the MEC toxicology dataset reflects both eligibility for medical-examiner investigation and the decision to obtain and/or report toxicology testing. The dataset, therefore, does not represent a uniformly tested statewide population, and differences in testing practices across jurisdictions cannot be excluded.

Deaths recorded between January 1, 2020, and December 31, 2023, were eligible for inclusion. The analytic dataset contained 61,234 medical examiner cases, with observations contributed by all 67 Florida counties. County-level case counts varied substantially. Because the dataset did not contain information identifying the laboratory, assay platform, testing protocol, analyte-specific testing status, limits of detection or quantification, or specimen-specific testing procedures, we could not determine whether individual substances were tested uniformly across all cases or whether testing practices differed among counties. Analyses, therefore, reflect substances documented as detected in the available medical examiner toxicology records. For the cohort selection process, see Figure 1.

**Figure 1:**
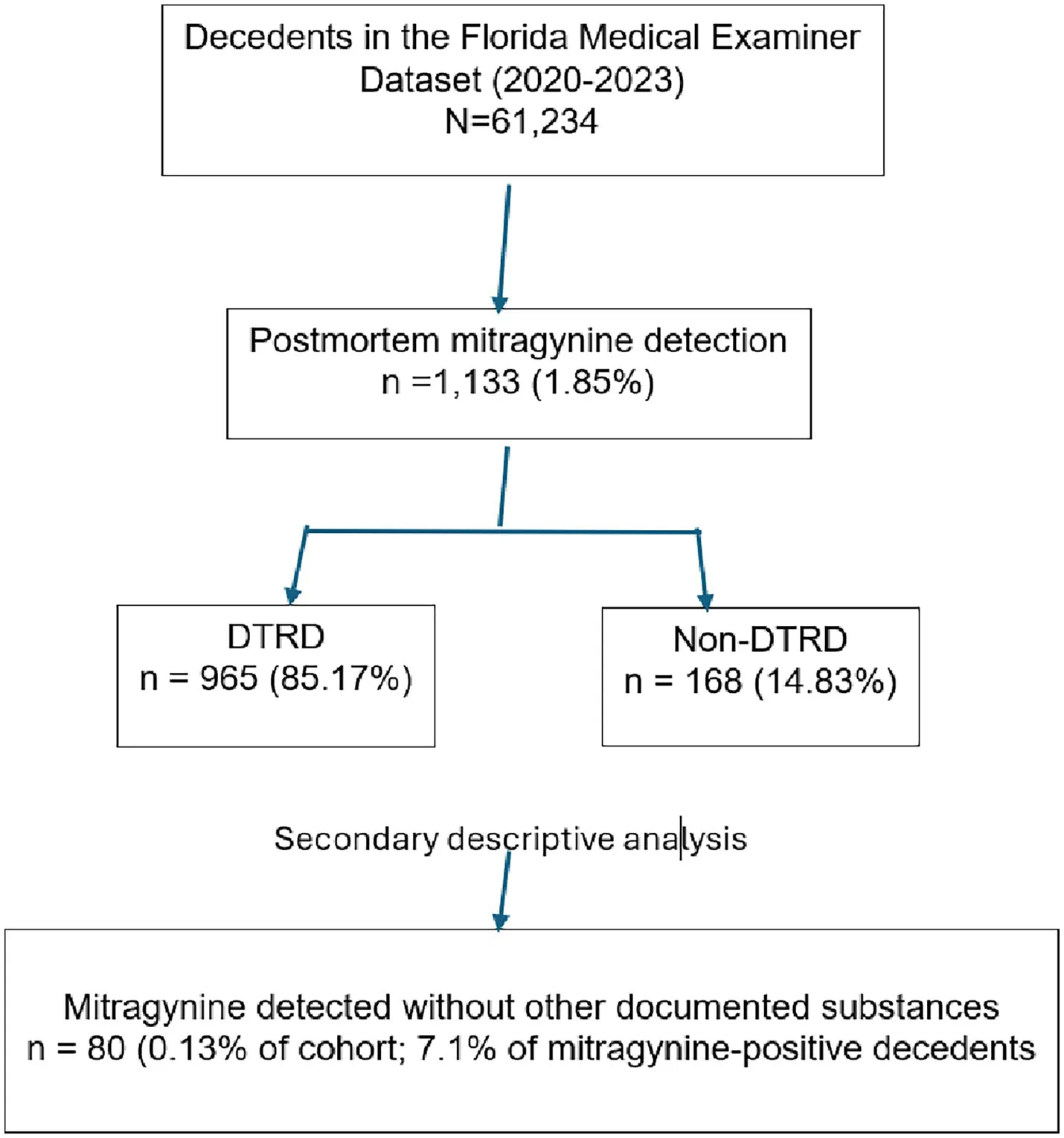
Flow diagram of cohort selection.

To contextualize the number of deaths represented in the MEC dataset, annual statewide mortality data for Florida were used as the denominator. Florida recorded 239,381 deaths in 2020, 261,246 in 2021, 238,953 in 2022, and 229,045 in 2023, for a total of 968,625 deaths during the study period (31). The proportion of deaths in which substances were identified through medical examiner toxicology reports was calculated by dividing the 61,234 deaths identified in the MEC dataset by the total number of deaths recorded statewide during 2020–2023.

### Toxicology exposures and analytical classification

The primary exposure of interest was mitragynine positivity, defined as a documented positive toxicology finding for mitragynine in the MEC dataset. Mitragynine was used as an analytical marker of kratom exposure because it is a principal alkaloid of kratom and a commonly measured forensic marker of kratom consumption. Importantly, detection of mitragynine was interpreted as evidence of toxicologically documented exposure and not as evidence that kratom caused the death.

The prespecified toxicology dataset included mitragynine, fentanyl and fentanyl analogs, heroin, morphine, oxycodone, methadone, buprenorphine, codeine, hydrocodone, hydromorphone, tramadol, benzodiazepines, cocaine, amphetamine, methamphetamine, cannabinoids and synthetic cannabinoids, alcohol, and xylazine. Benzodiazepines included alprazolam, clonazepam, diazepam, lorazepam, midazolam, nordiazepam, oxazepam, temazepam, and zolpidem. Substances outside this prespecified analytic classification were not included in the corresponding exposure analyses.

Substance exposure was coded as present when a positive toxicology finding was documented. Because the dataset did not provide case-level information regarding whether each analyte was tested in every decedent, a recorded negative finding was not necessarily interpreted as definitive evidence that a substance was absent. In particular, substances outside the prespecified panel, newly emerging synthetic opioids, novel psychoactive substances, adulterants, and other substances not included in the reported testing may have remained undetected. Consequently, “kratom-only” was defined operationally as mitragynine positivity without another substance documented in the prespecified dataset, rather than as definitive evidence of exposure to kratom alone.

The dataset did not contain quantitative mitragynine or 7-hydroxymitragynine concentrations, dose, timing of ingestion, route of administration, product formulation, source of kratom, or other exposure characteristics. Nor did it provide sufficient case-level information to establish the analytical platform, the limit of detection, the limit of quantification, or the confirmatory testing method. Therefore, the analysis could not assess concentration-response relationships or distinguish recent, substantial exposure from lower-level or more remote exposure.

### Sociodemographic and clinical covariates

Prespecified sociodemographic covariates included sex (male or female), race/ethnicity (White, Black or African American, Hispanic, Asian, and Other), age in years as a continuous variable, age category (0–17, 18–24, 25–34, 35–44, 45–54, 55–64, and ≥65 years), and county-level population density. Population density was categorized as large central metropolitan, large fringe metropolitan, medium metropolitan, small metropolitan, micropolitan, or noncore according to National Center for Health Statistics classifications based on population size and commuting patterns (39).

We additionally constructed a binary indicator of single-substance versus multiple-substance exposure based on the substances documented in the prespecified toxicology dataset.

Clinical conditions documented in the medical examiner’s record were grouped into cardiac, respiratory, hepatic, and renal categories and coded as binary variables (present/absent). Cardiac conditions included hypertensive heart disease, cardiovascular disease, atherosclerotic cardiovascular disease, acute cardiac dysrhythmia, left ventricular hypertrophy, cardiomegaly, congestive heart failure, coronary artery disease, and ischemic cardiomyopathy, among others. Respiratory conditions included chronic obstructive pulmonary disease, acute bronchopneumonia, acute respiratory distress syndrome, emphysema, pleural effusion, and COVID-19-related pulmonary findings, among others. Hepatic conditions included cirrhosis, acute or chronic hepatitis, hepatic failure, fatty liver changes, hepatic steatosis, liver fibrosis, intrahepatic abscess, and end-stage liver disease. Renal conditions included renal failure, end-stage renal disease, renal neoplasm, pyelonephritis, and polycystic kidney disease. Diabetes-related conditions included diabetes mellitus type II, diabetes, uncontrolled diabetes, and diabetic ketoacidosis, among others.

These conditions were prespecified because underlying organ dysfunction may alter drug absorption, distribution, metabolism, and elimination and may therefore influence the clinical consequences of substance exposure.

### Primary outcome

The primary outcome was drug toxicity–related death (DTRD), defined from the medical examiner’s recorded cause-of-death classification. DTRD was coded as a binary outcome (yes/no) when the recorded cause of death met the study’s prespecified criteria for drug toxicity or drug intoxication.

DTRD reflects the medical examiner’s determination that drug toxicity or intoxication contributed to the death; it does not establish mitragynine as the specific causal agent. This distinction is particularly important in decedents with multiple detected substances.

The outcome was evaluated among decedents with at least one substance documented in the available toxicology record. No assumptions were made regarding whether a detected substance was prescribed, illicit, intentionally used, misused, or causally responsible for the death. Consistent with the observational design, associations between mitragynine detection and DTRD were interpreted as epidemiologic associations rather than evidence of causation.

### Statistical analysis

Decedent characteristics were summarized according to mitragynine exposure status. Categorical variables were reported as frequencies and percentages and compared using Pearson’s χ^2^ test; continuous variables were summarized using means and standard deviations or medians and interquartile ranges, as appropriate.

Logistic regression was used to evaluate associations between toxicological exposures and drug toxicity. We first fitted a multivariable model examining the associations of mitragynine and co-occurring substance exposures with drug toxicity. A second, fully adjusted multivariable model additionally accounted for prespecified demographic characteristics, manner of death, multiple-drug exposure, and clinical conditions. Manner of death was included as a covariate to account for differences in the circumstances surrounding death that could influence the likelihood of drug toxicity. Results are presented as adjusted odds ratios (aORs) with 95% confidence intervals (CIs).

Opioid exposure definitions varied according to the objective of each analysis. Fentanyl was examined specifically in the mitragynine–opioid interaction analysis because it was the most frequently detected opioid in the study population and therefore represented the predominant opioid exposure in this dataset. For the opioid-burden sensitivity analysis, multiple opioid exposures were considered to capture the extent of concomitant opioid exposure rather than exposure to a single opioid. For the fentanyl-positivity sensitivity analysis, fentanyl and fentanyl analogs were grouped together to capture fentanyl-class exposure more comprehensively. These analysis-specific definitions were selected to address distinct analytic questions and to assess the robustness of the primary findings.

Several sensitivity and effect-modification analyses were performed. Interaction terms were used to evaluate whether the association between mitragynine exposure and drug toxicity differed according to opioid exposure or sex. Additional sensitivity analyses evaluated opioid burden and fentanyl positivity. For the opioid-burden analysis, burden was based on the number of fentanyl and fentanyl analogs detected because these represented the most frequently detected opioids in the study population. These analyses were conducted to assess the robustness of the primary findings and potential effect modification.

All analyses were conducted using Stata/SE version 17.0 (42). All statistical tests were two-sided, with *p* < 0.05 considered statistically significant.

## Results

### Study population and characteristics of mitragynine-positive decedents

A total of 61,234 decedents were included in the analysis, of whom 29,877 (48.79%) met the definition of a drug toxicity-related death (DTRD). The proportion of decedents classified as having DTRD varied across substances: mitragynine (kratom), 965 of 1,133 (85.17%); opioids, 24,516 of 31,480 (77.88%); cocaine, 11,388 of 15,185 (75.0%); stimulants, 8,807 of 11,994 (73.43%); benzodiazepines, 6,515 of 11,732 (55.53%); cannabinoids, 5,464 of 14,001 (39.03%); and alcohol, 8,941 of 24,617 (36.32%). Because individual decedents could have multiple substances detected, these percentages are not mutually exclusive.

Mitragynine was detected in 1,133 decedents (1.85% of the cohort). The mean age was 38.52 years (SD, 10.14), and most decedents were male (899 [79.42%]), White (1,078 [95.15%]), and residents of urban counties (1,113 [98.23%]). The 25–44-year age group accounted for the largest proportion of mitragynine-positive decedents (809 [71.40%]). Among mitragynine-positive decedents, 965 (85.17%) met criteria for DTRD, and 1,053 (93.0%) had multiple substances detected. Characteristics of the study population are presented in Table 1. The distribution of concomitantly detected substances among mitragynine-positive and mitragynine-negative decedents is shown in Table 2.

**Table 1:** Characteristics of kratom (mitragynine) exposures versus no kratom (mitragynine) exposures.

Characteristics	Kratom (mitragynine) users (*n* = 1,133)	Non-kratom users (*n* = 60,101)	*p*-values
Age, mean (SD)	38.52 (10.14)	46.33 (16.11)	
Age category (yrs), *n* (%)			<0.001
0–17	1 (0.09%)	786 (1.31%)	
18–24	60 (5.30%)	3,943 (6.56%)	
25–34	374 (33.01%)	11,264 (18.74%)	
35–44	435 (38.39%)	12,926 (21.51%)	
45–54	158 (13.95%)	11,554 (19.22%)	
55–64	94 (8.30%)	11,678 (19.43%)	
65+	11 (0.97%)	7,950 (13.23%)	
Sex, *n* (%)			<0.001
Male	899 (79.42%)	44,301 (73.72%)	
Female	233 (20.58%)	15,796 (26.28%)	
Race/Ethnicity, *n* (%)			<0.001
White	1,078 (95.23%)	46,507 (77.42%)	
Black	23 (2.03%)	10,319 (17.18%)	
Hispanic	27 (2.39%)	2,554 (4.25%)	
Asian	3 (0.27%)	287 (0.48%)	
Other	1 (0.09%)	401 (0.67%)	
Population density, *n* (%)			<0.001
Large Central Metro (Urban)	346 (30.54%)	21,300 (35.44%)	
Large Fringe Metro (Urban)	369 (32.57%)	15,169 (25.24%)	
Medium Metro (Urban)	349 (30.80%)	17,935 (29.84%)	
Small Metro (Urban)	49 (4.32%)	3,564 (5.93%)	
Micropolitan (Rural)	12 (1.06%)	1,210 (2.01%)	
Noncore (Rural)	8 (0.71%)	923 (1.54%)	
Manner of death			<0.001
Accident	989 (87.37%)	38,851 (64.65%)	
Homicide	17 (1.50%)	3,958 (6.59%)	
Natural cause	48 (4.24%)	8,808 (14.66%)	
Suicide	65 (5.74%)	7,559 (12.58%)	
Undetermined cause	13 (1.15%)	920 (1.53%)	
# of substance(s) use, *n* (%)			<0.001
Single drug use	80 (7.06%)	22, 096 (36.77%)	
Multidrug use	1,053 (92.94%)	38,004 (63.23%)	
Drug intoxication, *n* (%)	965 (85.17%)	28,912 (48.11%)	<0.001
Without drug intoxication	168 (14.83%)	31,189 (51.89%)	
Other substances			
Opioids	864 (76.26%)	30,616 (50.94%)	<0.001
No opioid exposure	269 (23.74%)	29,485 (49.06%)	
Cocaine	349 (30.80%)	14,836 (24.69%)	<0.001
No cocaine exposure	784 (69.20%)	45,265 (75.31%)	
Cannabinoids	197 (17.39%)	13,804 (22.97%)	<0.001
No cannabinoids	936 (82.61%)	46,297 (77.03%)	
Stimulant	225 (19.86%)	11,769 (19.58%)	0.82
No stimulant	908 (80.14%)	48,332 (80.42%)	
Alcohol	298 (26.30%)	24,319 (40.46%)	<0.001
No alcohol	835 (73.70%)	35,782 (59.54%)	
Benzodiazepine	332 (29.30%)	11,400 (18.97%)	<0.001
No benzodiazepine	801 (70.70%)	48,701 (81.03%)	
Clinical conditions			
Cardiac-related	143 (12.62%)	11,108 (18.48%)	<0.001
No cardiac-related conditions	990 (87.38%)	48,993 (81.52%)	
Respiratory	42 (3.71%)	4,178 (6.95%)	<0.001
No respiratory-related cond.	1,091 (96.29%)	55,914 (93.05%)	
Kidney-related conditions	2 (0.18%)	345 (0.57%)	0.08
No kidney-related conditions	1,131 (99.82%)	59,748 (99.43%)	
Liver-related conditions	14 (1.24%)	923 (1.54%)	0.42
No liver-related conditions	1,118 (98.76%)	59,171 (98.46%)	
Diabetes-related	5 (0.44%)	1,051 (1.75%)	0.001
No diabetes-related conditions	1,128 (99.56%)	59,050 (98.25%)	

**Table 2:** Co-occurrence of other drugs by categories among decedents with and without mitragynine exposure.

Drugs	Mitragynine exposures, *n* (%)	Non-mitragynine exposures, *n* (%)
Benzodiazepines		
Alprazolam	177 (3.18%)	5,397 (96.82%)
Clonazepam	108 (4.86%)	2,114 (95.14%)
Diazepam	46 (2.91%)	1,534 (97.09%)
Lorazepam	14 (1.20%)	1,155 (98.80%)
Midazolam	2 (0.18%)	1,092 (99.82%)
Nordiazepam	49 (2.95%)	1,614 (97.05%)
Oxazepam	26 (2.79%)	906 (97.21%)
Temazepam	35 (2.99%)	1,135 (97.01%)
Zolpidem	10 (1.70%)	579 (98.30%)
Opioids		
Buprenorphine	16 (1.72%)	912 (98.28%)
Codeine	24 (2.30%)	1,018 (97.70%)
Fentanyl	789 (3.29%)	23,191 (96.71%)
Fentanyl analogs	317 (4.76%)	6,346 (95.24%)
Heroin	62 (3.81%)	1,566 (96.19%)
Hydrocodone	38 (2.11%)	1763 (97.89%)
Hydromorphone	28 (1.75%)	1,572 (98.25%)
Methadone	20 (1.51%)	1,306 (98.49%)
Morphine	102 (2.36%)	4,225 (97.64%)
Oxycodone	91 (2.10%)	4,236 (97.90%)
Oxymorphone	49 (2.38%)	2012 (97.62%)
Tramadol	51 (2.88%)	1719 (97.12%)
Stimulants		
Amphetamine	168 (1.72%)	9,614 (98.28%)
Methamphetamine	171 (1.61%)	10,443 (98.39%)

### Mitragynine detection without other documented substances

Among the 61,234 decedents, 80 (0.13%) had postmortem mitragynine detected without any other substance captured by the available substance variables. These cases are described as *mitragynine-positive without other documented substances* rather than definitively “kratom-only,” because the available data cannot establish that all potentially relevant substances, including emerging or untested adulterants, were assessed in every case.

Within this subgroup, the mean age was 39.5 years (SD, 11.7); 60 decedents (75.0%) were male, 73 (91.3%) were White, and 77 (96.3%) were from urban counties. The most represented age groups were 35–44 years (30 [37.5%]) and 25–34 years (19 [23.8%]). Most deaths were classified as accidental (50 [62.5%]), and 43 (53.8%) were classified as DTRD with mitragynine listed as the substance causing the drug toxicity. Thus, these 43 cases represented 0.14% of all DTRDs and 4.5% of DTRDs among the 956 mitragynine-positive decedents classified as having DTRD. Cardiac conditions were documented in 16 cases (20.0%), respiratory conditions in 5 (6.3%), and liver-related conditions in 2 (2.5%); no kidney-related conditions were documented. Detailed characteristics are presented in Table 3.

**Table 3:** Characteristics of decedents with mitragynine detection only (*n* = 80).

Characteristics	*n*	%
Age, mean (SD)	39.52 (11.7)	
Age category		
0–17 years old	1	1.25
18–24 years old	5	6.25
25–34 years old	19	23.75
35–44 years old	30	37.5
45–54 years old	14	17.5
55–64 years old	11	13.75
65 + years old	0	0
Sex		
Male	60	75
Female	20	25
Race/ethnicity		
White	73	91.25
Black	3	3.75
Hispanic	2	2.5
Asian	2	2.5
Other	0	0
Population density		
Large Central Metro	30	37.5
Large Fringe Metro	14	17.5
Medium Metro	26	32.5
Small Metro	7	8.75
Micropolitan	2	2.5
Noncore	1	1.25
Manner of death		
Accidents	50	62.5
Homicide	1	1.25
Natural cause	14	17.5
Suicide	13	16.25
Undetermined	1	1.25
Drug Intoxication	43	53.75
No drug intoxication	37	46.25
Cardiac-related conditions	16	20
No cardiac-related conditions	64	80
Respiratory-related conditions	5	6.25
No respiratory-related conditions	75	93.75
Liver-related conditions	2	2.5
No liver-related conditions	78	97.5
Kidney-related conditions	0	0
No kidney-related conditions	80	100
Diabetes-related conditions	0	0
No diabetes-related conditions	80	100

### Associations between mitragynine detection and drug toxicity

#### Univariate analysis

In the initial univariate logistic regression model, which did not account for concomitant substance exposure, mitragynine-positive decedents had substantially higher odds of DTRD than mitragynine-negative decedents (OR, 6.20; 95% CI, 5.26–7.31; *p* < 0.001). Among the substances examined, opioid exposure demonstrated the strongest association with DTRD (OR, 16.02; 95% CI, 15.39–16.67; *p* < 0.001).

Alcohol and cannabinoid detection were associated with lower odds of DTRD in univariate analyses. Alcohol exposure was associated with 57% lower odds of DTRD (OR, 0.43; 95% CI, 0.41–0.44; *p* < 0.001), while cannabinoid exposure was associated with approximately 40% lower odds (OR, 0.60; 95% CI, 0.58–0.62; *p* < 0.001). These inverse associations should not be interpreted as evidence of protective effects, given the potential for confounding and differences in patterns of polysubstance exposure. Results are presented in Table 4.

**Table 4:** Multivariable logistic regression examining associations between co-occurring substance exposures and drug toxicity (*N* = 61,234).

Variable	aOR	95% CI	*p*-value
Mitragynine exposure	6.93	5.67–8.48	<0.001
Opioid exposure	16.44	15.70–17.23	<0.001
Cocaine exposure	4.96	4.71–5.23	<0.001
Benzodiazepine exposure	0.99	0.94–1.04	0.66
Stimulant exposure	4.8	4.52–5.09	<0.001
Alcohol exposure	1.12	1.07–1.18	<0.001
Cannabinoid exposure	0.75	0.71–0.79	<0.001

#### Multivariable analysis

Two multivariable logistic regression models were used to evaluate the association between mitragynine detection and DTRD. Model 1 included mitragynine and co-occurring substance exposures, whereas Model 2 additionally adjusted for demographic and health-related characteristics.

##### Model 1: Mitragynine and co-occurring substance exposures

After adjustment for other substance exposures, mitragynine detection remained strongly associated with DTRD (adjusted odds ratio [aOR], 6.93; 95% CI, 5.67–8.48; *p* < 0.001). Opioid exposure demonstrated the strongest association (aOR, 16.44; 95% CI, 15.70–17.23; *p* < 0.001), followed by cocaine (aOR, 4.96; 95% CI, 4.71–5.23; *p* < 0.001) and stimulant exposure (aOR, 4.80; 95% CI, 4.52–5.09; *p* < 0.001). Alcohol exposure was associated with a modest increase in the odds of DTRD (aOR, 1.12; 95% CI, 1.07–1.18; *p* < 0.001), whereas cannabis exposure was associated with lower odds (aOR, 0.75; 95% CI, 0.71–0.79; *p* < 0.001). Benzodiazepine exposure was not independently associated with DTRD (aOR, 0.99; 95% CI, 0.94–1.04; *p* = 0.663).

Model 1 demonstrated substantial explanatory power (likelihood-ratio χ^2^ = 30,708.36; *p* < 0.001; pseudo-R^2^ = 0.362), with opioid and mitragynine exposures showing the strongest associations with DTRD (Table 4).

##### Model 2: Mitragynine, substance exposures, and demographic and health factors

After additional adjustment for demographic and health-related factors, the association between mitragynine detection and DTRD remained significant but was attenuated (aOR, 4.23; 95% CI, 3.29–5.44; *p* < 0.001). Opioid exposure remained the strongest substance-related predictor (aOR, 9.91; 95% CI, 9.30–10.56; *p* < 0.001), followed by cocaine (aOR, 2.56; 95% CI, 2.40–2.73; *p* < 0.001) and stimulant exposure (aOR, 1.93; 95% CI, 1.78–2.08; *p* < 0.001).

Cannabis (aOR, 0.52; 95% CI, 0.49–0.56; *p* < 0.001), benzodiazepine (aOR, 0.88; 95% CI, 0.82–0.95; *p* = 0.001), and alcohol exposure (aOR, 0.74; 95% CI, 0.70–0.78; *p* < 0.001) were associated with lower odds of DTRD. These inverse associations may reflect confounding and differences in exposure patterns rather than protective effects.

Among demographic factors, age 65 years or older was associated with lower odds of DTRD (aOR, 0.59; 95% CI, 0.45–0.76; *p* > 0.001), whereas female sex was associated with higher odds (aOR, 1.29; 95% CI, 1.22–1.37; *p* < 0.001). Higher population density was associated with a modest increase in the odds of DTRD (aOR, 1.22; 95% CI, 1.13–1.30; *p* < 0.001).

Clinical conditions were also associated with drug toxicity after multivariable adjustment. Cardiac-related conditions (aOR, 1.27; 95% CI, 1.16–1.38) and liver-related conditions (aOR, 1.49; 95% CI, 1.13–1.96) were associated with higher odds of drug toxicity, whereas respiratory-related conditions (aOR, 0.59; 95% CI, 0.53–0.66), kidney disease (aOR, 0.39; 95% CI, 0.26–0.58), and diabetes (aOR, 0.51; 95% CI, 0.40–0.66) were associated with lower odds. These findings should be interpreted as adjusted associations rather than evidence of protective or harmful effects of individual clinical conditions.

In contrast, exposure to multiple drugs was associated with twofold higher odds of DTRD (aOR, 2.01; 95% CI, 1.88–2.17; *p* < 0.001). Full results are presented in Table 5.

**Table 5:** The associations of kratom (mitragynine) and co-variates* with drug toxicity using crude and multivariate logistic regression.

Covariates	Crude analysis (Univariate)			Adjusted analysis (Multivariate)		
	OR	95% CI	*p*-value	OR	95% CI	*p*-value
Mitragynine detected	6.2	5.26–7.31	<0.001	4.23	3.29–5.44	<0.001
Non-mitragynine detected (ref)						
Age Category (years)						
0–17 years old (ref)						
18–24 years old	2.28	1.89–2.76	<0.001	1.34	1.03–1.75	0.031
25–34 years old	5.35	4.46–6.41	<0.001	1.90	1.47–2.45	<0.001
35–44 years old	6.82	5.69–8.17	<0.001	2.22	1.72–2.86	<0.001
45–54 years old	5.06	4.22–6.07	<0.001	2.20	1.70–2.83	<0.001
55–64 years old	3.54	2.96–4.25	<0.001	1.90	1.48–2.46	<0.001
65 + years old	1.21	1.01–1.46	0.04	0.59	0.45–0.76	<0.001
Sex						
Male (ref)						
Female	1.29	1.24–1.34	<0.001	1.29	1.22–1.37	<0.001
Race/ethnicity						
White (ref)						
Black	0.51	0.49–0.54	<0.001	0.91	0.84–0.98	0.011
Hispanic	0.75	0.69–0.81	<0.001	0.80	0.71–0.91	0.001
Asian	0.47	0.37–0.60	<0.001	0.67	0.46–0.96	0.031
Other	0.73	0.60–0.89	0.002	0.62	0.46–0.84	0.002
Population density						
Large Central Metro (ref)						
Large Fringe Metro	1.32	1.2–1.37	<0.001	1.22	1.13–1.30	<0.001
Medium Metro	1.17	1.13–1.22	<0.001	1.40	1.31–1.50	<0.001
Small Metro	0.95	0.89–1.02	0.19	1.08	0.96–1.22	0.165
Micropolitan	0.7	0.62–0.79	<0.001	1.08	0.90–1.29	0.429
Noncore	0.54	0.47–0.62	<0.001	0.69	0.56–0.84	<0.001
Manner of death						
Accident (ref)						
Homicide	0.005	0.004–0.006	<0.001	0.007	0.005–0.009	<0.001
Natural cause	0.0003	0.0002–0.0007	<0.001	0.0004	0.0002–0.0008	<0.001
Suicide	0.073	0.069–0.078	<0.001	0.144	0.133–0.156	<0.001
Undetermined cause	0.136	0.117–0.158	<0.001	0.181	0.150–0.219	<0.001
Single drug exposure (ref)						
Multiple drug exposures	9.4	9.03–9.79	<0.001	2.02	1.88–2.17	<0.001
Other substances						
Alcohol use	0.43	0.41–0.44	<0.001	0.74	0.70–0.78	<0.001
Non-alcohol use (ref)						
Benzodiazepines	1.4	1.34–1.45	<0.001	0.88	0.82–0.95	0.001
Non-Benzodiazepines (ref)						
Cannabis	0.6	0.58–0.62	<0.001	0.52	0.49–0.56	<0.001
Non-cannabis (ref)						
Cocaine	4.47	4.29–4.66	<0.001	2.56	2.39–2.73	<0.001
Non-cocaine (ref)						
Opioid users	16.02	15.39–16.67	<0.001	9.91	9.30–10.56	<0.001
Non-opioid users (ref)						
Stimulants	3.69	3.53–3.86	<0.001	1.93	1.78–2.08	<0.001
Non-stimulant (ref)						
Clinical conditions						
Cardiac-related conditions	0.41	0.39–0.43	<0.001	1.27	1.16–1.38	<0.001
Non-cardiac related conditions (ref)						
Respiratory-related conditions	0.45	0.42–0.48	<0.001	0.59	0.53–0.66	<0.001
Non-respiratory related conditions (ref)						
Liver-related conditions	0.47	0.41–0.55	<0.001	1.49	1.13–1.96	0.005
Non-liver-related conditions (ref)						
Kidney-related conditions	0.29	0.22–0.37	<0.001	0.39	0.26–0.58	<0.001
Non-kidney related conditions (ref)						
Diabetes-related	0.239	0.204–0.279	<0.001	0.51	0.40–0.66	<0.001
Non-diabetes-related conditions (ref)						

^*^Significant demographic covariates.

All variance inflation factor values were below 2.1 (maximum, 2.04; mean, 1.22), indicating no evidence of problematic multicollinearity.

#### Effect modification by sex

In the adjusted effect-modification analysis, mitragynine exposure was strongly associated with drug toxicity-related death among both males and females. Among males, mitragynine exposure was associated with 4.49-fold higher odds of drug toxicity-related death (adjusted OR [aOR], 4.49; 95% CI, 3.56–5.66). The corresponding association among females was similar (aOR, 4.16; 95% CI, 2.70–6.43). Among decedents without mitragynine exposure, females had modestly higher odds of drug toxicity-related death than males (aOR, 1.16; 95% CI, 1.10–1.22) (Table 6). However, there was no evidence of statistically significant effect modification by sex (mitragynine × female sex interaction aOR, 0.93; 95% CI, 0.57–1.52), indicating that the association between mitragynine exposure and drug toxicity-related death did not differ significantly between males and females (see Figure 2).

**Table 6:** Association between mitragynine exposure and drug toxicity-related death by sex: adjusted effect modification analysis.

Variable	Adjusted OR (95% CI)
Mitragynine exposure among males	4.49 (3.56–5.66)
Female vs. male among decedents without mitragynine exposure	1.16 (1.10–1.22)
Mitragynine x female sex interaction	0.93 (0.57–1.52)
Mitragynine exposure among females	4.16 (2.70–6.43)

**Figure 2:**
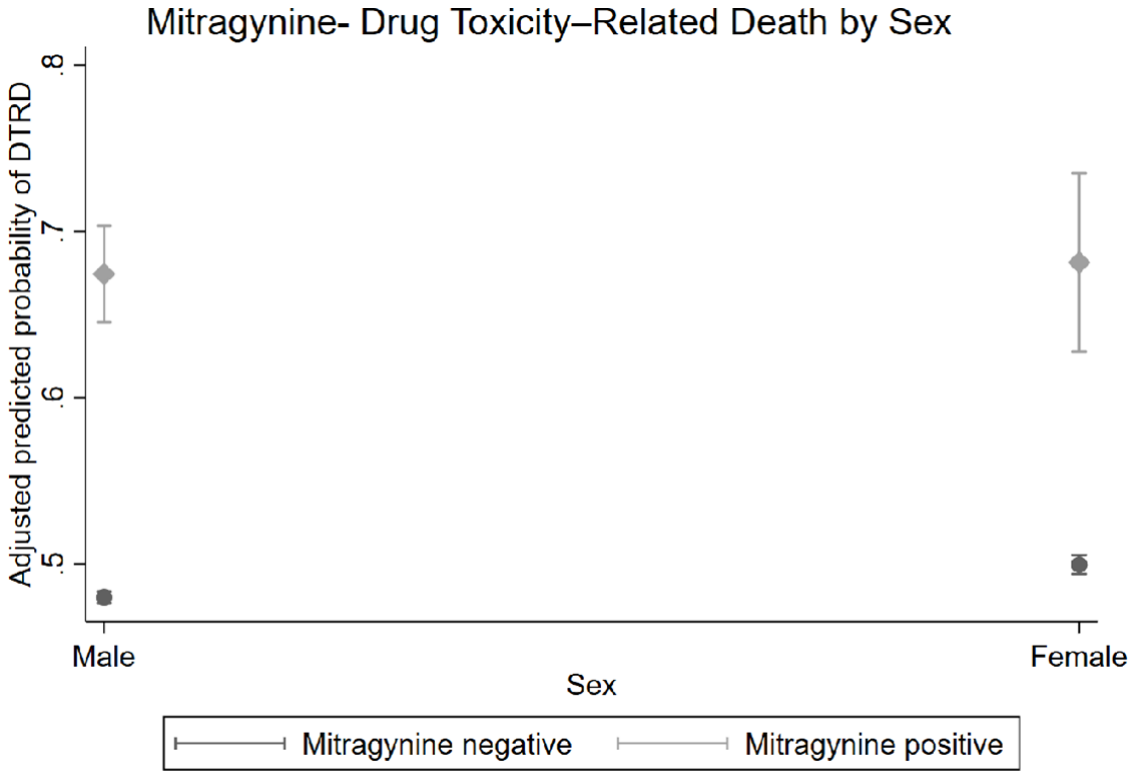
Mitragynine-DTRD association by sex. Adjusted predicted probabilities of drug toxicity-related death according to mitragynine detection and sex, derived from the fully adjusted logistic regression model with a mitragynine-by-sex interaction term. Error bars represent 95% confidence intervals. There was no statistically significant evidence of effect modification by sex (interaction OR, 0.93; 95% CI, 0.57–1.52; *p* = 0.767).

#### Model performance

The fully adjusted model demonstrated strong discrimination, with an area under the receiver operating characteristic curve (AUC) of 0.891. Overall predictive accuracy was good, with a Brier score of 0.133 compared with 0.250 for the null model, corresponding to a 46.7% reduction in the Brier score (see Figure 3).

**Figure 3:**
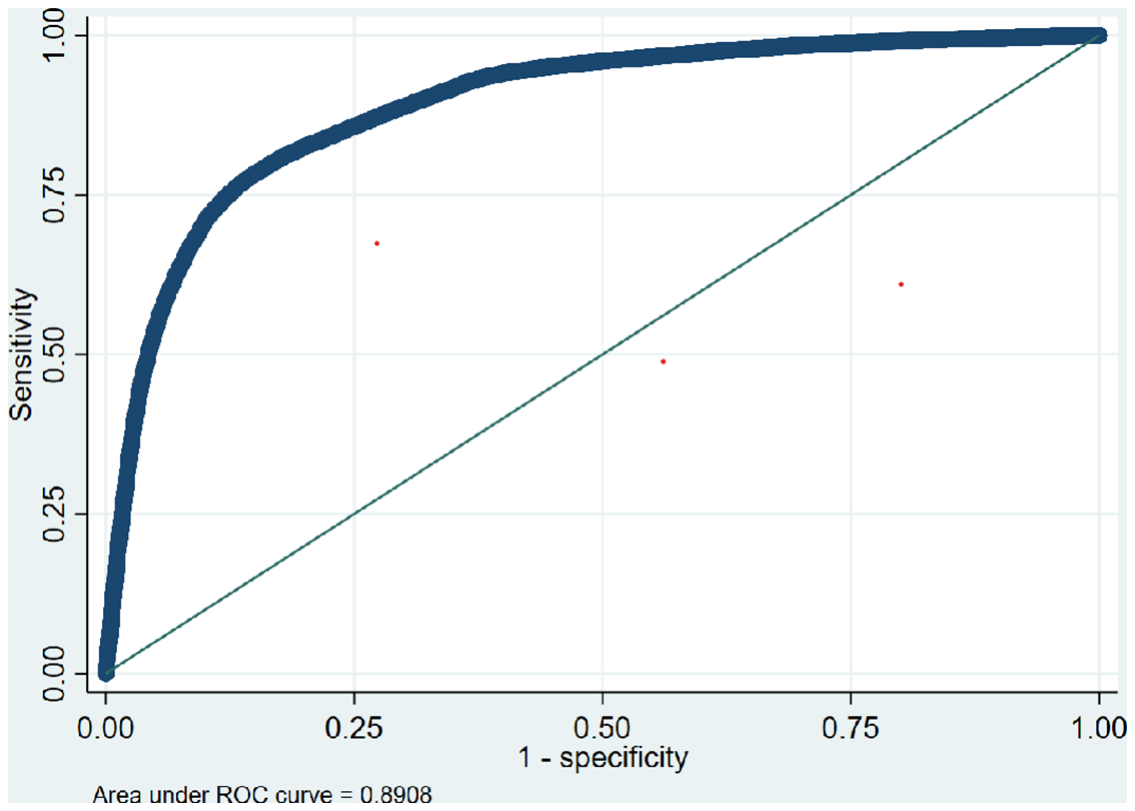
Receiver operating characteristic curve demonstrating discrimination of the fully adjusted model for drug toxicity-related death. The area under the receiver operating characteristic curve (AUC) was 0.891, indicating strong discrimination of the fully adjusted model for drug toxicity-related death.

Calibration was also favorable, with a calibration intercept of 0.00 (95% CI, −0.023 to 0.023) and a calibration slope of 1.00 (95% CI, 0.986–1.014). The calibration plot showed generally close agreement between predicted and observed probabilities across risk deciles, although modest departures from the ideal calibration line were observed in selected risk groups (Figure 4). The Hosmer–Lemeshow goodness-of-fit test was statistically significant (χ^2^₈ = 402.42; *p* < 0.001), indicating detectable departures from perfect fit. Given the large sample size, this result was interpreted in conjunction with the calibration plot, calibration intercept, and slope, and Brier score rather than in isolation.

**Figure 4:**
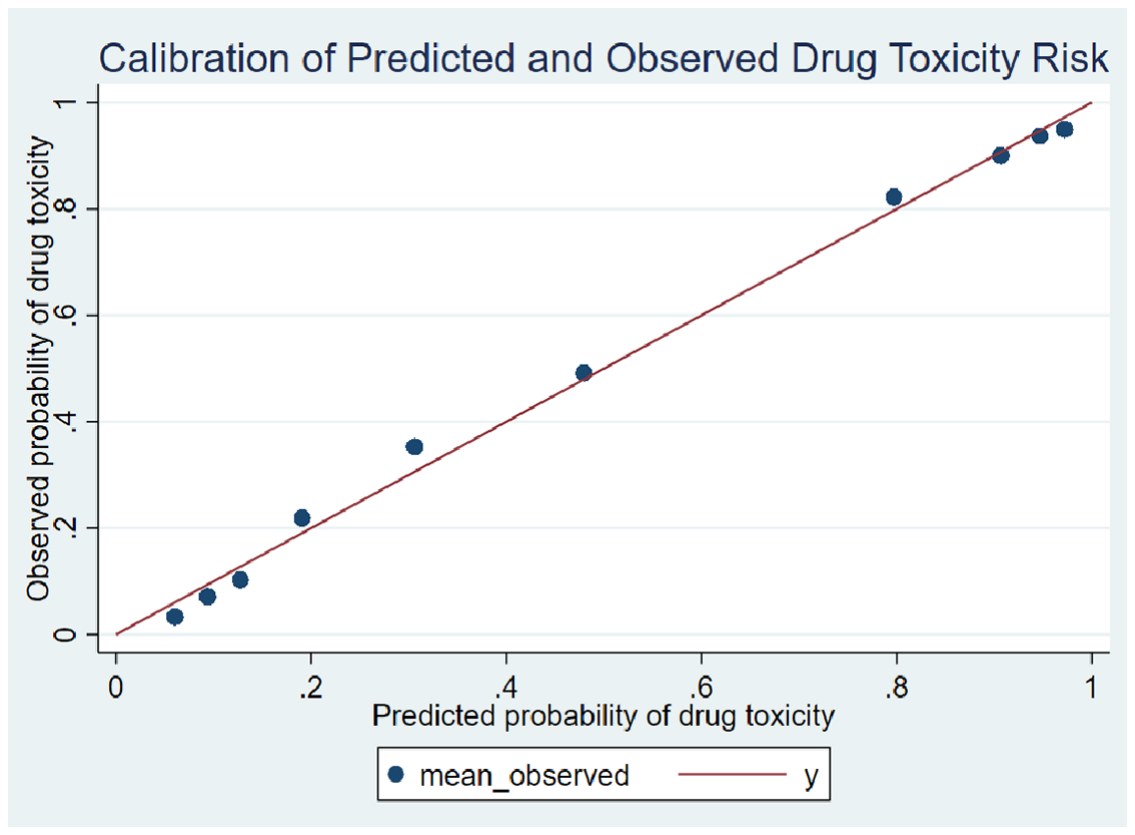
Calibration of the fully adjusted model for predicted and observed drug toxicity-related death risk observed versus predicted probabilities of drug toxicity-related death across risk deciles in the fully adjusted logistic regression model. The diagonal line represents perfect calibration; points represent observed event probabilities. The model demonstrated good calibration, with a calibration intercept of 0.00 (95% CI, 0.023 to 0.023), a calibration slope of 1.00 (95% CI, 0.986–1.014), and a Brier score of 0.133. The Hosmer-Lemeshow test was statistically significant (x^2^8 = 402.42; *p* < 0.001), indicating some departure from perfect fit in this large study population.

#### Mitragynine–opioid interaction

We evaluated whether fentanyl exposure modified the association between mitragynine detection and DTRD. There was no evidence of multiplicative interaction between mitragynine and fentanyl exposure (interaction OR, 0.90; 95% CI, 0.54–1.49; *p* = 0.690).

On the additive scale, the adjusted predicted probability of DTRD was 29.2% among decedents exposed to neither mitragynine nor fentanyl, 82.3% among those exposed to fentanyl only, 54.2% among those exposed to mitragynine only, and 93.3% among those exposed to both substances. The additive interaction measure was −0.140 (95% CI, −0.193 to −0.087), and the relative excess risk due to interaction (RERI) was −0.479 (95% CI, −0.662 to −0.295), providing no evidence of positive additive interaction.

#### County-adjusted sensitivity analysis

To assess whether geographic heterogeneity across Florida counties influenced the observed association, we performed a sensitivity analysis adjusting for county as a categorical variable. The analytic sample comprised 61,198 decedents with complete data for the regression model. After county adjustment, the association between mitragynine detection and DTRD remained robust (OR, 3.83; 95% CI, 3.10–4.73), compared with an OR of 3.69 (95% CI, 2.93–4.65) in the corresponding fully adjusted model without county adjustment. Thus, adjustment for county-level differences did not materially alter the association between mitragynine detection and DTRD.

#### Sensitivity analyses for opioid burden

To assess whether the association between mitragynine detection and DTRD was attributable to concomitant opioid exposure, we performed an additional multivariable analysis incorporating the number of distinct opioids detected as a categorical measure of opioid burden. After adjustment for opioid burden, benzodiazepine, stimulant, cocaine, cannabis, and alcohol exposure; age; sex; race/ethnicity; population density; multiple-drug exposure; and clinical conditions, mitragynine detection remained independently associated with DTRD (aOR, 4.73; 95% CI, 3.85–5.82; *p* < 0.001).

Increasing opioid burden was strongly associated with DTRD. Compared with decedents without opioid exposure, the adjusted odds of DTRD increased with the number of distinct opioids detected: aOR, 10.26 (95% CI, 9.70–10.85) for 1 opioid; 13.19 (95% CI, 12.20–14.27) for 2; 17.02 (95% CI, 15.04–19.27) for 3; 26.83 (95% CI, 21.93–32.82) for 4; 27.81 (95% CI, 19.31–40.04) for 5; and 56.68 (95% CI, 26.00–123.55) for 6 opioids. All associations were statistically significant (*p* < 0.001).

In a separate sensitivity analysis incorporating cumulative opioid exposure, mitragynine detection remained strongly associated with DTRD (aOR, 5.54; 95% CI, 4.59–6.68; *p* < 0.001). The odds of DTRD increased progressively with increasing opioid exposure, from 11.95 (95% CI, 11.43–12.49) among decedents with 1 opioid exposure to 82.92 (95% CI, 38.76–177.37) among those with 6 or more opioid exposures (all *p* < 0.001). The overall model demonstrated substantial explanatory power (likelihood-ratio χ^2^ = 23,350.26; *p* < 0.001; pseudo-R^2^ = 0.275). Detailed results are presented in Table 7.

**Table 7:** Multivariable logistic regression analysis of the association between mitragynine exposure, cumulative opioid exposure, and drug toxicity (*n* = 61,234).

Drug Toxicity	OR	95% CI	*p*-value
Mitragynine			
Exposure	5.54	4.59–6.68	<0.001
With 1 opioid	11.95	11.43–12.49	<0.001
2 opioids	18.16	17.08–19.3	<0.001
3 opioids	22.8	20.45–25.43	<0.001
4 opioids	36.24	29.99–43.79	<0.001
5 opioids	41.01	28.91–58.16	<0.001
6 + opioids	82.92	38.76–177.37	<0.001

#### Sensitivity analysis by fentanyl positivity

We further examined whether the association between mitragynine detection and DTRD differed according to fentanyl or fentanyl-analog positivity. Among decedents with fentanyl/fentanyl-analog positivity (*n* = 23,972), mitragynine detection remained significantly associated with DTRD (aOR, 3.23; 95% CI, 2.06–5.07; *p* < 0.001). The association was also significant among fentanyl-negative decedents (*n* = 37,226; aOR, 4.61; 95% CI, 3.63–5.86; *p* < 0.001). The mitragynine-by-fentanyl interaction was not statistically significant (χ^2^ = 0.73; *p* = 0.393), indicating no evidence that fentanyl positivity modified the association between mitragynine detection and DTRD.

## Discussion

In this statewide retrospective analysis of 61,234 decedents represented in the Florida medical examiner toxicology dataset, postmortem mitragynine detection was associated with substantially higher odds of drug toxicity-related death (DTRD) after adjustment for demographic, clinical, and polysubstance variables. The association remained evident after accounting for co-occurring substance exposures and was attenuated but persisted after further adjustment for demographic and health-related factors. Mitragynine detection was also associated with DTRD in sensitivity analyses incorporating opioid burden and among both fentanyl-positive and fentanyl-negative decedents. However, mitragynine-positive deaths without other documented substances were uncommon. These findings suggest that mitragynine detection identifies a subgroup of decedents with a high burden of drug toxicity, particularly within complex patterns of polysubstance exposure, but they do not establish that mitragynine independently caused or contributed pharmacologically to the fatal event.

### Comparison with previous literature

In this cohort, approximately 85% of mitragynine-positive decedents were classified as having DTRD, and more than 90% had multiple substances detected. The high prevalence of polysubstance exposure underscores the difficulty of attributing fatal toxicity to any single substance and is consistent with the broader literature describing kratom exposure in the context of concomitant psychoactive substance use. Previous studies have largely relied on case reports, poison-center data, or relatively small cohorts. Smallets et al. (32), for example, reported 35 deaths among 55 mitragynine-confirmed cases identified through toxicological screening, with 26 cases involving mitragynine-associated drug toxicity. Post et al. (13) identified 11 deaths among 1,807 kratom exposures reported to the National Poison Data System between 2011 and 2017, whereas Peran et al. (33) described 10 nonfatal, self-reported kratom overdose cases in the Czech Republic between 2019 and 2022, most of which involved concurrent substance use. Differences in the magnitude of these findings likely reflect differences in study populations, case ascertainment, exposure definitions, and outcome classification. In contrast to poison-center and self-report data, the present study used postmortem toxicological information from a large statewide medical examiner population and therefore provides a broader characterization of mitragynine detection among decedents undergoing toxicological evaluation.

The demographic profile of mitragynine-positive decedents, predominantly middle-aged, male, White, and residing in urban counties, was broadly consistent with previous epidemiological observations, including those reported by Post et al. (13) and Olsen et al. (12). The observed male predominance is consistent with broader epidemiological evidence demonstrating sex-related differences in unintentional drug-overdose mortality, although these patterns vary by race and other sociodemographic characteristics (38). The concentration of mitragynine-positive cases among individuals aged 25–44 years also parallels demographic patterns observed in populations affected by opioid and stimulant use. These similarities provide contextual support for the observed demographic distribution, while also highlighting the extent to which kratom exposure occurs within populations already affected by substance-use-related morbidity and mortality.

### Interpretation of the mitragynine association

Across the multivariable models, mitragynine remained associated with DTRD. In the model adjusting for co-occurring substance exposures, mitragynine detection was associated with approximately sevenfold higher odds of DTRD, whereas the fully adjusted model yielded an association of approximately fivefold higher odds. The attenuation after adjustment for demographic and health-related factors suggests that part of the crude association may be explained by differences in the characteristics of mitragynine-positive and mitragynine-negative decedents. Nevertheless, the persistence of the association after extensive adjustment indicates that mitragynine detection remains an important marker of DTRD within this selected population.

An important alternative explanation is that mitragynine detection may function, at least in part, as a marker of underlying substance-use severity rather than as an independent determinant of fatal toxicity. Individuals with more severe opioid or polysubstance use may be more likely to use kratom, including as an attempt to reduce, replace, or self-manage their use of other substances. Kratom has been described as a harm-reduction strategy among some individuals with substance use disorders (3, 34). Consequently, the higher frequency of DTRD among mitragynine-positive decedents could partly reflect differences in underlying risk, including severity of opioid use, patterns of polysubstance exposure, treatment history, or other characteristics not captured in the dataset.

The associations observed for clinical conditions warrant cautious interpretation. Cardiac- and liver-related conditions were associated with higher odds of drug toxicity, whereas respiratory conditions, kidney disease, and diabetes were associated with lower odds. These findings do not necessarily indicate that individual comorbidities increase or decrease biological susceptibility to drug toxicity. Differences in disease severity, healthcare utilization, medication and substance-use patterns, competing causes of death, and unmeasured clinical factors may contribute to these associations. In addition, because the study was restricted to decedents investigated within the medical examiner and toxicology system, these estimates should not be interpreted as population-level effects of comorbid disease on the risk of drug toxicity.

Although the present models incorporated multiple measured co-exposures and demographic and clinical characteristics, residual and unmeasured confounding cannot be excluded. In particular, the available data do not provide detailed information on dose, frequency, route of administration, timing of ingestion, severity or chronicity of substance use, or treatment history. Thus, the observed association should not be interpreted as evidence that mitragynine independently causes fatal toxicity. Rather, mitragynine detection may represent a combination of potential pharmacologic contribution and underlying characteristics of a population at elevated risk for drug toxicity.

The observation that 80 decedents had mitragynine detected, with no other substance captured by the available substance variables, provides additional context. Only 43 of these cases were classified as DTRD, representing a small proportion of all DTRDs and of mitragynine-positive DTRDs. Importantly, these cases cannot be considered definitively “kratom-only” deaths because the dataset does not establish that every potentially relevant substance, including emerging adulterants, was tested for or detected. Accordingly, the relatively small number of mitragynine-positive decedents without other documented substances limits the extent to which the present data can distinguish an independent effect of mitragynine from toxicity associated with polysubstance exposure.

### Role of opioids and polysubstance exposure

As expected, opioid exposure demonstrated the strongest association with DTRD across the models (OR 15.43, 95% CI 14.80–16.08, *p* < 0.001), followed by cocaine and other stimulants. These findings are consistent with prior work, including Henningfield et al. (35) and Olsen et al. (12), highlighting the substantial contribution of opioid-involved exposure to drug-related mortality. Torrico et al. (36) similarly reported four cases involving kratom exposure among 214 opioid-overdose deaths in California in 2020. Together, these findings reinforce the importance of considering kratom within the broader context of opioid and polysubstance use rather than as an isolated exposure. This broader context is particularly important given evidence that overdose mortality risk varies across age groups and birth cohorts, with generational patterns evolving alongside changes in drug availability and the wider overdose epidemic (40).

The sensitivity analyses further demonstrated a strong relationship between cumulative opioid burden and DTRD. The odds of DTRD increased progressively with the number of distinct opioids detected, exceeding 80-fold among individuals with six or more opioid exposures in one analysis. Importantly, the association between mitragynine detection and DTRD persisted after adjustment for cumulative opioid exposure. These findings suggest that the observed association with mitragynine was not fully explained by the number of opioids detected. Nevertheless, the strong opioid dose–response pattern also emphasizes that opioid exposure remains a dominant determinant of drug toxicity in this population.

The findings, therefore, should not be interpreted as indicating that kratom has a risk magnitude comparable with opioids. Rather, the approximately fivefold association observed for mitragynine in the fully adjusted model was substantially smaller than the association observed for opioid exposure. This distinction is important when interpreting the clinical and public health implications of mitragynine detection.

The inverse associations observed for alcohol and cannabis should likewise be interpreted cautiously. In particular, alcohol was inversely associated with DTRD in the unadjusted analysis but showed a weaker association after adjustment for other substances and covariates. These findings are unlikely to represent protective pharmacological effects. Instead, they may reflect differences in polysubstance-use patterns, exposure ascertainment, selection effects, or the composition of the populations in which alcohol, cannabis, and high-potency opioids are detected. For example, individuals with alcohol or cannabis exposure may differ systematically from those with high-risk opioid or stimulant exposure. The stronger inverse association observed for alcohol in the univariate model (OR 0.43) compared with the fully adjusted model (OR 0.87) further supports the possibility that confounding by co-exposure patterns contributed substantially to the crude association.

### Mitragynine–fentanyl interaction

An important clinical and toxicological question is whether concomitant mitragynine and opioid exposure is associated with greater-than-expected toxicity. In the present analysis, there was no evidence of positive interaction between mitragynine and fentanyl on either the multiplicative or additive scale. The interaction OR was 0.90 (95% CI, 0.54–1.49), while the additive interaction measure was −0.140 (95% CI, −0.193 to −0.087) and the RERI was −0.479 (95% CI, −0.662 to −0.295). These findings do not support a synergistic or potentiating association between mitragynine and fentanyl for the DTRD outcome examined.

The absence of statistical interaction should not, however, be interpreted as evidence that concomitant mitragynine and fentanyl exposure is safe or that mitragynine protects against opioid toxicity. Interaction analyses are dependent on the exposure and outcome definitions, the underlying model, and the absence of substantial residual confounding. Moreover, the dataset lacks quantitative concentrations, dose, timing, and route of exposure, all of which may be important determinants of pharmacologic interaction. The absence of observed interaction, therefore, indicates only that we did not detect evidence of effect modification under the conditions examined.

### Interpretation of sex effect modification

The findings suggest that the association between mitragynine exposure and drug toxicity-related death is substantial and broadly consistent across sexes. Although the point estimate was slightly lower among females than males (aOR, 4.16 vs. 4.49), the interaction estimate was close to the null (aOR, 0.93; 95% CI, 0.57–1.52), with the confidence interval encompassing 1, providing no statistical evidence that sex modifies the association. Thus, the observed association between mitragynine exposure and drug toxicity-related death appears to be relatively similar in males and females. The modestly higher odds observed among females in the absence of mitragynine exposure may reflect underlying differences in the characteristics of drug toxicity-related deaths between sexes rather than differential susceptibility to mitragynine-associated toxicity. Importantly, the absence of statistically significant interaction should not be interpreted as proof of identical effects across sexes; rather, the data do not provide sufficient evidence that the magnitude of the association differs by sex.

### Biological plausibility and clinical implications

From a pharmacological perspective, a potential contribution of kratom to drug toxicity is biologically plausible. Kratom constituents have activity at opioid, serotonergic, and adrenergic receptors and may influence central nervous system function. Accordingly, concomitant exposure to kratom and other psychoactive or sedating substances could plausibly affect respiratory, neurologic, or cardiovascular function. However, biological plausibility alone cannot establish causality, particularly in a population characterized by extensive polysubstance exposure.

The persistence of the association between mitragynine detection and DTRD across multiple adjusted models and sensitivity analyses is compatible with a potential pharmacologic contribution, but it is equally important to recognize that mitragynine may serve as a marker of a higher-risk substance-use phenotype. The absence of quantitative postmortem mitragynine concentrations, dose information, timing of ingestion, route of administration, and product-specific composition prevents assessment of whether detected mitragynine concentrations were sufficient to produce clinically meaningful pharmacologic effects at the time of death.

Clinically, these findings support heightened awareness of kratom exposure when evaluating patients with polysubstance use or suspected drug toxicity. They do not, however, support attributing a fatal event to kratom solely based on a positive postmortem mitragynine result. Toxicological interpretation should consider the complete pattern of detected substances, concentrations when available, circumstances of death, clinical history, and established causes of mortality.

From a public health perspective, the findings support continued surveillance of mitragynine-positive deaths, particularly as kratom use becomes more prevalent and products may contain variable concentrations of active constituents or other substances. Integrated harm-reduction and risk-mitigation strategies should address kratom within the broader landscape of opioid and polysubstance exposure rather than treating it as an isolated exposure.

### Strengths and limitations

This study has several strengths. First, it includes a large statewide cohort of 61,234 decedents and provides a systematic characterization of postmortem mitragynine detection within a medical examiner’s toxicology population. Second, the analysis incorporated multiple substance exposures as well as demographic and clinical characteristics, allowing assessment of whether the association between mitragynine and DTRD persisted after accounting for important potential confounders. Third, sensitivity analyses incorporating opioid burden, fentanyl positivity, and county-level adjustment provided additional assessment of the robustness of the findings. Finally, assessment of multicollinearity demonstrated low variance inflation factors, supporting the stability of the multivariable models despite substantial overlap among substance exposures.

Several limitations warrant consideration. First, the retrospective observational design precludes causal inference. Residual confounding is possible because important factors, including substance-use severity, dose, frequency, route, timing of exposure, specific kratom formulation or product, treatment history, and other clinical characteristics, were unavailable. The association between mitragynine detection and DTRD may therefore reflect a combination of direct or interactive toxicologic effects and characteristics of individuals with higher-risk patterns of substance use.

The second limitation concerns the analytical methods available for the sensitive detection and identification of mitragynine and 7-hydroxymitragynine in biological specimens (37). Second, mitragynine exposure was defined by postmortem detection rather than by quantitative concentration. The available dataset contained information indicating whether mitragynine was detected in postmortem urine but did not provide quantitative concentrations of mitragynine or 7-hydroxymitragynine. A positive urine result establishes prior exposure but does not establish the dose, recency, magnitude, or pharmacologic activity of exposure at the time of death. Mitragynine may therefore have been present at concentrations that were incidental, residual, or pharmacologically relevant, and the available data cannot distinguish among these possibilities. The absence of quantitative measurements also precludes evaluation of a concentration–response relationship.

Third, the dataset does not contain sufficient information to characterize specific kratom products, formulations, adulterants, or contaminants. This is particularly important because commercial kratom products may differ in alkaloid composition and may contain additional substances. Consequently, the findings cannot be generalized to all kratom products or interpreted as evidence of toxicity attributable specifically to mitragynine rather than another component of a kratom-containing product.

Fourth, selection of decedents into the medical examiner and toxicology-tested population may introduce selection bias. The study dataset does not represent all deaths occurring in Florida; rather, it consists of deaths investigated within the medical examiner system for which toxicology information was available. The decision to conduct toxicological testing may depend on the circumstances of death, medical history, suspected substance involvement, age, and other factors. Conditioning the analysis on inclusion in this selected population may therefore introduce selection or collider bias, potentially inducing or amplifying associations between mitragynine detection and DTRD. The findings should consequently be interpreted as associations within the Florida medical examiner toxicology population and should not be generalized directly to all Florida decedents or the general population.

Fifth, the outcome definition reflects medical examiner and toxicological classification rather than a determination that mitragynine was the primary cause of death. In a polysubstance setting, attribution of causality to a single substance is inherently challenging. Although mitragynine or kratom has been identified in reported fatal cases, there is insufficient evidence to establish a reliable population-level estimate of the frequency with which mitragynine directly causes or contributes to death. The frequent presence of co-occurring opioids, stimulants, cocaine, benzodiazepines, and other substances further limits the ability to isolate the contribution of mitragynine to individual deaths. Moreover, detection of mitragynine in postmortem toxicology does not establish that it directly caused or contributed to death. Thus, our findings should be interpreted as an association between mitragynine detection and DTRD rather than evidence of direct mitragynine-attributable mortality. Future studies incorporating quantitative mitragynine and 7-hydroxymitragynine concentrations, comprehensive toxicological profiles, clinical histories, and circumstances surrounding death are needed to better characterize the causal contribution of mitragynine to fatal toxicity.

Sixth, the available clinical variables provide limited information regarding the timing, severity, and chronicity of comorbid conditions. Associations between cardiac, respiratory, renal, and hepatic conditions and DTRD may therefore reflect differences in healthcare utilization, exposure patterns, competing causes of death, or other forms of selection rather than true protective or harmful effects. These findings should not be interpreted as evidence that such conditions biologically reduce the risk of drug toxicity. In addition, the lack of information regarding mental health history and prior substance use is an important limitation. Variables such as underlying psychiatric conditions, previous substance use, tolerance, and suicidal ideation were not available and therefore could not be incorporated into the analysis. These factors may influence patterns of substance exposure, risk-taking behaviors, and the clinical consequences of exposure, potentially confounding the observed associations. Consequently, the findings should be interpreted with consideration of these unmeasured factors. Future studies incorporating more comprehensive mental health and substance use histories may provide additional insight into the factors associated with the outcomes examined in this study.

Finally, missing data and the use of complete-case analyses may have introduced additional bias, although the extent of missingness was limited. The relatively small number of individuals at the highest levels of opioid exposure also resulted in wider confidence intervals in these strata. These limitations reinforce the need for prospective or carefully designed population-based studies incorporating detailed exposure histories and quantitative toxicology.

### Implications for screening, prevention, and future research

Examining drug detection patterns in drug-related compared with non-drug-related deaths may help identify substance-exposure patterns that warrant greater attention in clinical and public health screening and prevention efforts. In this study, the association between mitragynine detection and drug toxicity, particularly in the context of frequent opioid and polysubstance exposure, suggests that mitragynine and commonly co-occurring substances may warrant consideration when evaluating individuals at risk for substance-related toxicity. These findings may help inform targeted toxicology testing, clinical risk assessment, harm-reduction counseling, and prevention strategies, particularly among individuals with polysubstance exposure. Future studies should move beyond binary detection of mitragynine and incorporate quantitative concentrations of mitragynine and 7-hydroxymitragynine, dose and timing information, route of administration, specific product composition, and comprehensive toxicological profiles. Linking toxicology results with clinical records, circumstances of death, prescribing information, and longitudinal substance-use histories may help distinguish mitragynine exposure as a marker of high-risk substance use from a direct contributor to toxicity. Appropriately selected comparison populations will also be important; in particular, comparisons of mitragynine-positive and mitragynine-negative decedents with similar patterns of opioid and polysubstance exposure may provide a more informative assessment of the association between mitragynine and fatal toxicity. Prospective clinical and pharmacokinetic studies are also needed to establish clinically relevant concentrations, characterize potential interactions with opioids and other psychoactive substances, and determine whether specific kratom formulations are associated with differential risk. Because the present study is observational, these findings should be viewed as informing potential targets for screening and prevention rather than demonstrating the effectiveness of any specific intervention.

## Conclusion

In this large statewide analysis of decedents represented in the Florida medical examiner toxicology dataset, postmortem mitragynine detection was associated with higher odds of DTRD after adjustment for multiple demographic, clinical, and substance-related factors. The association persisted across sensitivity analyses incorporating opioid burden, county adjustment, and fentanyl positivity, while mitragynine-positive deaths without other documented substances were uncommon. Opioid exposure remained the dominant substance-related correlate of DTRD, and no evidence of positive multiplicative or additive interaction between mitragynine and fentanyl was observed.

These findings should be interpreted as associations within a selected medical examiner and toxicology-tested population and should not be interpreted as evidence that mitragynine causes DTRD or as estimates of the risk associated with kratom use in the general population. The observed association may reflect direct or interactive toxicologic effects, underlying substance-use severity, or residual and unmeasured confounding. Continued toxicological surveillance of mitragynine-positive deaths is warranted to characterize trends and co-exposure patterns, while future studies incorporating quantitative toxicology, detailed exposure histories, product-specific information, and appropriate comparison populations are needed to determine whether and to what extent mitragynine contributes to drug toxicity–related mortality.

## Data Availability

Publicly available datasets were analyzed in this study. The raw data came from the Florida Department of Law Enforcement from 2020 to 2023 and is publicly available upon request from the FDLE.
