# Supplementary material for: Association of postmortem mitragynine positivity with fatal drug toxicity in polysubstance exposures—a restrospective analysis
Source: Front Public Health. 2026 Oct 30;14:1923369. doi: 10.3389/fpubh.2026.1923369 (PMC13759796; doi:10.3389/fpubh.2026.1923369)
Supplement: Supplementary file 1 [file Table_1.DOCX]

Appendices

Table 1. Characteristics of Kratom (Mitragynine) Exposures versus No Kratom (Mitragynine) Exposures.

| Characteristics | Kratom (Mitragynine) users (n=1,133) | Non-Kratom users  (n= 60,101) | P-Values |
| --- | --- | --- | --- |
| Age, mean (SD) | 38.52 (10.14) | 46.33 (16.11) |  |
| Age Category (yrs), n (%)  0-17  18-24  25-34  35-44  45-54  55-64  65+ | 1 (0.09%)  60 (5.30%)  374 (33.01%)  435 (38.39%)  158 (13.95%  94 (8.30%)  11 (0.97%) | 786 (1.31%)  3,943 (6.56%)  11,264 (18.74%)  12,926 (21.51%)  11,554 (19.22%)  11,678 (19.43%)  7,950 (13.23%) | <0.001 |
| Sex, n (%)  Male  Female | 899 (79.42%)  233 (20.58%) | 44,301 (73.72%)  15,796 (26.28%) | <0.001 |
| Race/Ethnicity, n (%)  White  Black  Hispanic  Asian  Other | 1,078 (95.23%)  23 (2.03%)  27 (2.39%)  3 (0.27%)  1 (0.09%) | 46,507 (77.42%)  10,319 (17.18%)  2,554 (4.25%)  287 (0.48%)  401 (0.67%) | <0.001 |
| Population Density, n (%)  Large Central Metro (Urban)  Large Fringe Metro (Urban)  Medium Metro (Urban)  Small Metro (Urban)  Micropolitan (Rural)  Noncore (Rural) | 346 (30.54%)  369 (32.57%)  349 (30.80%)  49 (4.32%)  12 (1.06%)  8 (0.71%) | 21,300 (35.44%)  15,169 (25.24%)  17,935 (29.84%)  3,564 (5.93%)  1,210 (2.01%)  923 (1.54%) | <0.001 |
| # of Substance(s) Use, n (%)  Single drug use  Multidrug use | 80 (7.06%)  1,053 (92.94%) | 22, 096 (36.77%)  38,004 (63.23%) | <0.001 |
| Drug intoxication, n (%)  Without drug intoxication  ***Other substances:***  Opioids  No opioid exposure  Cocaine  No cocaine exposure  Cannabinoids  No cannabinoids  Stimulant  No stimulant  Alcohol  No alcohol  Benzodiazepine  No benzodiazepine  ***Clinical conditions:***  Cardiac-related  No cardiac-related conditions  Respiratory  No respiratory-related cond.  Kidney-related conditions  No kidney-related conditions  Liver-related conditions  No liver-related conditions | 965 (85.17%)  168 (14.83%)  864 (76.26%)  269 (23.74%)  349 (30.80%)  784 (69.20%)  197 (17.39%)  936 (82.61%)  225 (19.86%)  908 (80.14%)  298 (26.30%)  835 (73.70%)  332 (29.30%)  801 (70.70%)  143 (12.62%)  990 (87.38%)  42 (3.71%)  1,091 (96.29%)  2 (0.18%)  1,131 (99.82%)  14 (1.24%)  1,118 (98.76%) | 28,912 (48.11%)  31,189 (51.89%)  30,616 (50.94%)  29,485 (49.06%)  14,836 (24.69%)  45,265 (75.31%)  13,804 (22.97%)  46,297 (77.03%)  11,769 (19.58%)  48,332 (80.42%)  24,319 (40.46%)  35,782 (59.54%)  11,400 (18.97%)  48,701 (81.03%)  11,108 (18.48%)  48,993 (81.52%)  4,178 (6.95%)  55,914 (93.05%)  345 (0.57%)  59,748 (99.43%)  923 (1.54%)  59,171 (98.46%) | <0.001  <0.001  <0.001  <0.001  0.82  <0.001  <0.001  <0.001  <0.001  0.08  0.42 |
|  |  |  |  |

Table 2. Characteristics of Decedents with Mitragynine exposures only (n=80)

| **Characteristics** | **n** | **%** |
| --- | --- | --- |
| Age, mean (SD) | 39.52 (11.7) | |
| Age category |  |  |
| 0-17 years old | 1 | 1.25 |
| 18-24 years old | 5 | 6.25 |
| 25-34 years old | 19 | 23.75 |
| 35-44 years old | 30 | 37.5 |
| 45-54 years old | 14 | 17.5 |
| 55-64 years old | 11 | 13.75 |
| 65+ years old | 0 | 0 |
| Sex |  |  |
| Male | 60 | 75 |
| Female | 20 | 25 |
| Race/ethnicity |  |  |
| White | 73 | 91.25 |
| Black | 3 | 3.75 |
| Hispanic | 2 | 2.5 |
| Asian | 2 | 2.5 |
| Other | 0 | 0 |
| Population Density |  |  |
| Large Central Metro | 30 | 37.5 |
| Large Fringe Metro | 14 | 17.5 |
| Medium Metro | 26 | 32.5 |
| Small Metro | 7 | 8.75 |
| Micropolitan | 2 | 2.5 |
| Noncore | 1 | 1.25 |
| Manner of Death |  |  |
| Accidents | 50 | 62.5 |
| Homicide | 1 | 1.25 |
| Natural Cause | 14 | 17.5 |
| Suicide | 13 | 16.25 |
| Undetermined | 1 | 1.25 |
| Drug Intoxication | 43 | 53.75 |
| No drug intoxication | 37 | 46.25 |
| Cardiac-related conditions | 16 | 20 |
| No cardiac-related conditions | 64 | 80 |
| Respiratory-related conditions | 5 | 6.25 |
| No respiratory-related conditions | 75 | 93.75 |
| Liver-related conditions | 2 | 2.5 |
| No liver-related conditions | 78 | 97.5 |
| Kidney-related conditions | 0 | 0 |
| No kidney-related conditions | 80 | 100 |

Table 3. The Associations of Kratom (mitragynine) and co-variates* with drug toxicity using crude and multivariate logistic regression

| Covariates | Crude Analysis (Univariate) | | | Adjusted Analysis (Multivariate) | | |
| --- | --- | --- | --- | --- | --- | --- |
|  | OR | 95% CI | *P*-value | OR | 95% CI | *P*-value |
| Mitragynine exposed | 6.2 | 5.26-7.31 | <0.001 | 4.65 | 3.79-5.72 | <0.001 |
| Non-mitragynine exposed (ref) |  |  |  |  |  |  |
| Age (continuous) | 0.98 | .981-.983 | <0.001 | 0.983 | 0.981-0.984 | <0.001 |
| Sex |  |  |  |  |  |  |
| Male (ref) |  |  |  |  |  |  |
| Female | 1.29 | 1.24-1.34 | <0.001 | 1.16 | 1.1-1.22 | <0.001 |
| Race/ethnicity |  |  |  |  |  |  |
| White (ref) |  |  |  |  |  |  |
| Black | 0.51 | .49-.54 | <0.001 | 0.68 | .64-.72 | <0.001 |
| Hispanic | 0.75 | .69-.81 | <0.001 | 0.84 | .75-.94 | 0.002 |
| Asian | 0.47 | .37-.60 | <0.001 | 0.61 | .44-.84 | 0.003 |
| Other | 0.73 | .60-.89 | 0.002 | 0.7 | .54-.93 | 0.012 |
| Population Density |  |  |  |  |  |  |
| Large Central Metro (ref) |  |  |  |  |  |  |
| Large Fringe Metro | 1.32 | 1.2-1.37 | <0.001 | 1.2 | 1.13-1.27 | <0.001 |
| Medium Metro | 1.17 | 1.13-1.22 | <0.001 | 1.34 | 1.27-1.42 | <0.001 |
| Small Metro | 0.95 | .89-1.02 | 0.19 | 0.996 | .91-1.1 | 0.94 |
| Micropolitan | 0.7 | .62-.79 | <0.001 | 1.1 | .94-1.29 | 0.25 |
| Noncore | 0.54 | .47-.62 | <0.001 | 0.7 | .58-.83 | <0.001 |
| Single drug exposure (ref) |  |  |  |  |  |  |
| Multiple drug exposures | 9.4 | 9.03-9.79 | <0.001 | 2.06 | 1.94-2.19 | <0.001 |
| Other substances: |  |  |  |  |  |  |
| Alcohol use | 0.43 | .41-.44 | <0.001 | 0.87 | .82-.91 | <0.001 |
| *Non-alcohol use (ref)* |  |  |  |  |  |  |
| Benzodiazepines | 1.4 | 1.34-1.45 | <0.001 | 0.8 | .76-.85 | <0.001 |
| *Non-Benzodiazepines (ref)* |  |  |  |  |  |  |
| Cannabis | 0.6 | .58-.62 | <0.001 | 0.51 | .48-.54 | <0.001 |
| *Non-cannabis (ref)* |  |  |  |  |  |  |
| Cocaine | 4.47 | 4.29-4.66 | <0.001 | 4.19 | 3.96-4.44 | <0.001 |
| *Non-cocaine (ref)* |  |  |  |  |  |  |
| Opioid users | 16.02 | 15.39-16.67 | <0.001 | 11.28 | 10.69-11.91 | <0.001 |
| *Non-opioid users (ref)* |  |  |  |  |  |  |
| Stimulants | 3.69 | 3.53-3.86 | <0.001 | 2.83 | 2.65-3.02 | <0.001 |
| *Non-stimulant (ref)* |  |  |  |  |  |  |
| Clinical conditions: |  |  |  |  |  |  |
| Cardiac-related conditions | 0.41 | .39-.43 | <0.001 | 0.58 | .54-.61 | <0.001 |
| *Non-cardiac related conditions* (ref) |  |  |  |  |  |  |
| Respiratory-related conditions | 0.45 | .42-.48 | <0.001 | 0.52 | .54-.61 | <0.001 |
| *Non-respiratory related conditions (ref)* |  |  |  |  |  |  |
| Liver-related conditions | 0.47 | .41-.55 | <0.001 | 0.86 | .72-1.02 | 0.09 |
| *Non-liver-related conditions (ref)* |  |  |  |  |  |  |
| Kidney-related conditions | 0.29 | .22-.37 | <0.001 | 0.37 | .27-.51 | <0.001 |
| *Non-kidney related conditions (ref)* |  |  |  |  |  |  |

*Significant demographic co-variates

Table 4. Multivariable Logistic Regression Examining Associations Between Co-occurring Substance Exposures and Drug Toxicity (N=61,234)

| Variable | aOR | 95% CI | P-Value |
| --- | --- | --- | --- |
| Mitragynine exposure | 6.93 | 5.67-8.48 | <0.001 |
| Opioid exposure | 16.44 | 15.70-17.23 | <0.001 |
| Cocaine exposure | 4.96 | 4.71-5.23 | <0.001 |
| Benzodiazepine exposure | 0.99 | 0.94-1.04 | 0.66 |
| Stimulant exposure | 4.8 | 4.52-5.09 | <0.001 |
| Alcohol exposure | 1.12 | 1.07-1.18 | <0.001 |
| Cannabinoid exposure | 0.75 | 0.71-0.79 | <0.001 |

Table 5. Association Between Mitragynine Exposure and Drug Toxicity

with Effect Modification by Gender

| **Variable** |  | **OR (95% CI)** | ***P* value** |
| --- | --- | --- | --- |
| Mitragynine exposure (ref: no) |  | 6.91 (5.73–8.33) | <0.001 |
| Gender (Female vs ref: Male) |  | 1.31 (1.26–1.35) | <0.001 |
| Mitragynine × Gender |  | 0.66 (0.44–0.98) | 0.039 |

Table 6. Multivariable logistic regression analysis of the association between

mitragynine exposure, cumulative opioid exposure, and drug toxicity (n = 61,234).

| **Drug Toxicity** | **OR** | **95% CI** | ***P*-value** |
| --- | --- | --- | --- |
| Mitragynine  Exposure | 5.54 | 4.59-6.68 | <0.001 |
| With 1 opioid | 11.95 | 11.43-12.49 | <0.001 |
| 2 opioids | 18.16 | 17.08-19.3 | <0.001 |
| 3 opioids | 22.8 | 20.45-25.43 | <0.001 |
| 4 opioids | 36.24 | 29.99-43.79 | <0.001 |
| 5 opioids | 41.01 | 28.91-58.16 | <0.001 |
| 6+ opioids | 82.92 | 38.76-177.37 | <0.001 |
